# A novel semi-dominant mutation in *brassinosteroid signaling kinase1* increases stomatal density

**DOI:** 10.3389/fpls.2024.1377352

**Published:** 2024-04-02

**Authors:** Eigo Ando, Kyomi Taki, Takamasa Suzuki, Toshinori Kinoshita

**Affiliations:** ^1^ Division of Biological Science, Graduate School of Science, Nagoya University, Nagoya, Aichi, Japan; ^2^ Institute of Transformative Bio-Molecules, Nagoya University, Nagoya, Aichi, Japan; ^3^ Department of Biological Chemistry, College of Bioscience and Biotechnology, Chubu University, Kasugai, Aichi, Japan

**Keywords:** *Arabidopsis thaliana*, brassinosteroid signaling, whole-mount immunohistochemistry, InsituPro, genetic screening, next-generation sequencing

## Abstract

Stomata play a pivotal role in balancing CO_2_ uptake for photosynthesis and water loss via transpiration. Thus, appropriate regulation of stomatal movement and its formation are crucial for plant growth and survival. Red and blue light induce phosphorylation of the C-terminal residue of the plasma membrane (PM) H^+^-ATPase, threonine, in guard cells, generating the driving force for stomatal opening. While significant progress has been made in understanding the regulatory mechanism of PM H^+^-ATPase in guard cells, the regulatory components for the phosphorylation of PM H^+^-ATPase have not been fully elucidated. Recently, we established a new immunohistochemical technique for detecting guard-cell PM H^+^-ATPase phosphorylation using leaves, which was expected to facilitate investigations with a single leaf. In this study, we applied the technique to genetic screening experiment to explore novel regulators for the phosphorylation of PM H^+^-ATPase in guard cells, as well as stomatal development. We successfully performed phenotyping using a single leaf. During the experiment, we identified a mutant exhibiting high stomatal density, *jozetsu* (*jzt*), named after a Japanese word meaning ‘talkative’. We found that a novel semi-dominant mutation in BRASSINOSTEROID SIGNALING KINASE1 (BSK1) is responsible for the phenotype in *jzt* mutant. The present results demonstrate that the new immunohistochemical technique has a wide range of applications, and the novel mutation would provide genetic tool to expand our understanding of plant development mediated by brassinosteroid signaling.

## Introduction

Stomata consist of pairs of guard cells on the plant epidermis and function to balance CO_2_ uptake for photosynthesis and water loss via transpiration. Stomatal movement (opening and closing) is regulated in response to various environmental and endogenous signals, including light (blue and red light)/darkness, CO_2_, humidity, the phytohormone abscisic acid (ABA), and drought stress (Roelfsema et al., 2012; Inoue and Kinoshita, 2017; Jezek and Blatt, 2017; Lawson and Matthews, 2020). Stomatal development is also influenced by environment and endogenous stimuli, such as light, CO_2_, phytohormones (ABA, auxin, and brassinosteroids), temperature, and water/osmotic stress (Qi and Torii, 2018; Han et al., 2021).

Light-induced stomatal opening is driven by the activation of the plasma membrane (PM) H^+^-ATPase in guard cells, which generates an electrochemical gradient across the PM to promote ion influx and a consequent turgor increase through osmotic water uptake by the cell (Shimazaki et al., 2007; Inoue and Kinoshita, 2017; Fuglsang and Palmgren, 2021; Palmgren, 2023). The activation of PM H^+^-ATPase is mediated by the phosphorylation of its C-terminal penultimate residue, threonine (Thr), in guard cells (Kinoshita and Shimazaki, 1999, 2002). Previous studies have identified regulators for blue light-induced phosphorylation of PM H^+^-ATPase in guard cells (Kinoshita et al., 2011; Takemiya et al., 2013a, 2013b; Hayashi et al., 2017). Although a PM-localized protein kinase insensitive to a potent protein kinase inhibitor, K-252a, is suggested to phosphorylate the penultimate Thr in PM H^+^-ATPase (Hayash et al., 2010), it has not been identified. Moreover, photosynthesis-dependent phosphorylation and clade D type2C protein phosphatase-mediated dephosphorylation of PM H^+^-ATPase (Ando and Kinoshita, 2018; Wong et al., 2021; Akiyama et al., 2022; Ando et al., 2022) raise a hypothesis that unidentified regulators are involved in the above processes in guard cells. Note that PM H^+^-ATPase trafficking in guard cells regulated by environmental stress greatly affects its activity and stomatal movement (Xue et al., 2018; Xia et al., 2019; Baena et al., 2024).

Stomatal development is a sequential process of cell division and differentiation. In *Arabidopsis thaliana* (Arabidopsis), stomatal development begins with a subset of protodermal cells called meristemoid mother cells, which undergo several asymmetric cell divisions to generate meristemoid cells surrounded by stomatal lineage ground cells. Subsequently, the meristemoid cells differentiate into guard mother cells (GMCs). Finally, GMCs undergo a symmetrical division, resulting in the formation of a pair of guard cells (Qi and Torii, 2018; Han et al., 2021). These processes are regulated by three basic helix-loop-helix (bHLH) transcription factors: SPEACHLESS (SPCH; MacAlister et al., 2007), MUTE (Pillitteri et al., 2007), and FAMA (Ohashi-Ito and Bergmann, 2006), as well as bHLH-leucin zipper proteins SCREAME(SCRM)/INDUCER OF CBF EXPRESSION1 and SCRM2 (Kanaoka et al., 2008). The stability of the stomatal bHLH proteins is regulated by the mitogen-activated protein kinase (MAPK) cascade, which includes YODA (YDA), MKK4/5/7/9, and MPK3/6 (Bergmann et al., 2004). The SPCH/SCRM heterodimer is responsible for the initiation and proliferation of the stomatal lineage through asymmetric divisions. The MUTE/SCRM dimer halts the asymmetric division mediated by SPCH/SCRM and induces the differentiation of meristemoids into GMCs. The FAMA/SCRM complex facilitates the last symmetric division and restricts further cell division in guard cells.

A phytohormone brassinosteroid (BR) coordinates plant growth and development (Zhu et al., 2013). PM-localized leucin rich repeat-receptor kinase (LRR-RK) BRASSINOSTEROID INSENSITIVE1 (BRI1) functions as a BR receptor (Li and Chory, 1997; Kinoshita et al., 2005). BR binding activates BRI1 kinase activity, involving the recruitment of the coreceptor kinase BRI1-ASSOCIATED RECEPTOR KINASE1 (BAK1; Li et al., 2002; Nam and Li, 2002), dissociation from inhibitory proteins (Wang and Chory, 2006), and transphosphorylation between BRI1 and BAK1 (Wang et al., 2008). Activated BRI1 phosphorylates substrate proteins, including BRASSINOSTEROID SIGNALLING KINASE1 (BSK1; Tang et al., 2008). The phosphorylated BSK1 induces BRI1-SUPPRESSOR1-mediated inactivation of GSK3-like kinase BRASSIONSTEROID INSENSITIVE2 (BIN2) to regulate downstream transcriptional regulation by BRASSINAZOLE RESISTANT1 (BZR1; He et al., 2002; Wang et al., 2002; Yin et al., 2002; Kim et al., 2011). In Arabidopsis leaves, BR has been shown to negatively regulate stomatal development. *bri1* and dominant *bin2* mutations result in a clustered stomata phenotype, whereas a mutation in *BZR1* does not affect stomatal development (Kim et al., 2012). Additionally, BIN2 has been shown to inhibit YDA activity (Kim et al., 2012). Thus, BIN2 is considered to regulate the MAPK cascade to control downstream stomatal transcription factors (Qi and Torii, 2018).

BSK1 is one of the members of receptor-like cytoplasmic kinase sub-family (RLCK-XII), which is consisted of 12 members in Arabidopsis (BSK1–BSK12; Tang et al., 2008). BSK proteins consist of the N-terminal kinase domain and the C-terminal tetratricopeptide repeats. BSK proteins do not contain a transmembrane region; however, myristoylation is suggested to enable their association with the PM, which may be required for their functions (Tang et al., 2008). The BSK family proteins exhibit functional redundancy, and genetic analysis of single mutants failed to show clear morphological defects, except for *bsk3*, which exhibits sensitivity to the BR biosynthesis inhibitor and insensitivity to brassinolide (Tang et al., 2008). Sreeramulu et al. (2013) indicated that knock-out of at least three BSK genes, including *bsk3*, is required for the morphological defects and altered response to exogenous brassinolide.

We previously established an immunohistochemical detection method for the guard-cell PM H^+^-ATPase and its phosphorylation status of the penultimate threonine. In this method, leaves instead of isolated epidermis or guard cells, were used as materials (Ando and Kinoshita, 2018, 2019). The use of isolated epidermis or guard cells often limits the applicability of conventional methods. Therefore, the new method employing leaves was designed to overcome these restrictions and have a broader range of applications. To demonstrate this, we carried out a genetic screening experiment as a model study. Genetic screening generally involves handling a single leaf, often from a dwarf plant, making it challenging to obtain a sufficient amount of epidermal tissue. Utilizing a commercial liquid-handling robot, we successfully performed simultaneous sample preparation and phenotyping for multiple samples. During the experiment, we identified a mutant with high stomatal density. Further analysis revealed that the mutation responsible for the phenotype is a novel semi-dominant allele of BSK1. Our mutant serves as a genetic tool to elucidate the function of BSK1 protein in plant development.

## Materials and methods

### Plant materials and growth conditions

*Arabidopsis thaliana* was used as experimental material. Ethyl methanesulphonate (EMS)-mutagenesis using *phot2-1* was carried out previously (Lightner and Caspar, 1998), and M_2_ population was used for screening. Columbia-0 (Col-0) was used as control plant for *bsk1* (SAIL_140_C04) and DNA extraction. The T-DNA insertion was confirmed by genomic PCR using primer sets shown in **Supplementary Table S1**. Landsberg *erecta* (L*er*) was used for a map-based cloning experiment. Plants were grown on soil under fluorescent lamps in the growth room. Photon flux densities, day length, temperature, and relative humidity was approximately 50 µmol m^−2^ s^−1^, 16-h, 20–24°C, and 40–60%, respectively.

### Immunohistochemical screening for stomatal traits

Previously developed immunohistochemical technique using leaves (Ando and Kinoshita, 2018) was employed as screening tool wherein phosphorylation of the penultimate residue of PM H^+^-ATPase (Thr) in guard cells was visualized. Leaves were collected from each M_2_ plants, then they were illuminated with red light (150 µmol m^−2^ s^−1^) and blue light (50 µmol m^−2^ s^−1^) simultaneously for 15 min. Illuminated leaves were fixed and attached to a microscope slide as described previously. Liquid handling including tissue permeabilization, blocking, antibody application, and washing the material steps was automated by Insitu Pro VSi (Intavis). Observed phenotype was confirmed using the remained leaves from the same M_2_ plants if possible, and validated in M_3_ plants.

### Stomatal density and index

For measurement of stomatal density and index, leaves were fixed and cleared according to the previous study (Kang et al., 2009). Six images per leaf were obtained, then the density and index were calculated on each image. Arithmetic mean of the six images was calculated as a representative value for the corresponding leaf. Data represent arithmetic means of the representative values obtained from at least three leaves with standard deviations.

### Next-generation sequencing

F_2_ plants were obtained by crossing EMS mutant line and its progenitor and those exhibiting mutant phenotype were selected and DNA was extracted from them in a bulk with the High pure PCR template preparation kit (Roche) according to the manufacturer’s instructions. The bulk population genomic DNA was subjected to whole-genome sequencing and analyzed by Mitsucal for mapping and identification of a mutation as described previously (Suzuki et al., 2018). Reanalysis of the mutation by sanger sequencing was performed using a primer set shown in **Supplementary Table S1**.

### Construction of plasmid vectors and transformation of plants

Genomic fragment of *BSK1* was amplified by nested PCR from Col-0 using primer sets shown in **Supplementary Table S1**. The fragment including −1,650 bp to +3,742 bp of the start codon was fused to pCAMBIA1300 digested with EcoRI using In-Fusion HD Cloning kit (Clontech). *Agrobacterium tumefaciens* (GV3101) was transformed with the construct, then used for generation of the transgenic plants.

### Statistical analyses

Statistical comparison of means was conducted using R software (R Core Team, 2023). Student’s *t* test or Dunnett’s test using *multcomp* package (Hothorn et al., 2008) was carried out for two independent means or multiple means with single control, respectively. Segregation ratio was analyzed by chi-square goodness of fit test. *P* < 0.05 was considered statistically significant.

## Results

### Genetic screening based on the immunohistochemical visualization of guard-cell PM H^+^-ATPase

**Figure 1:**
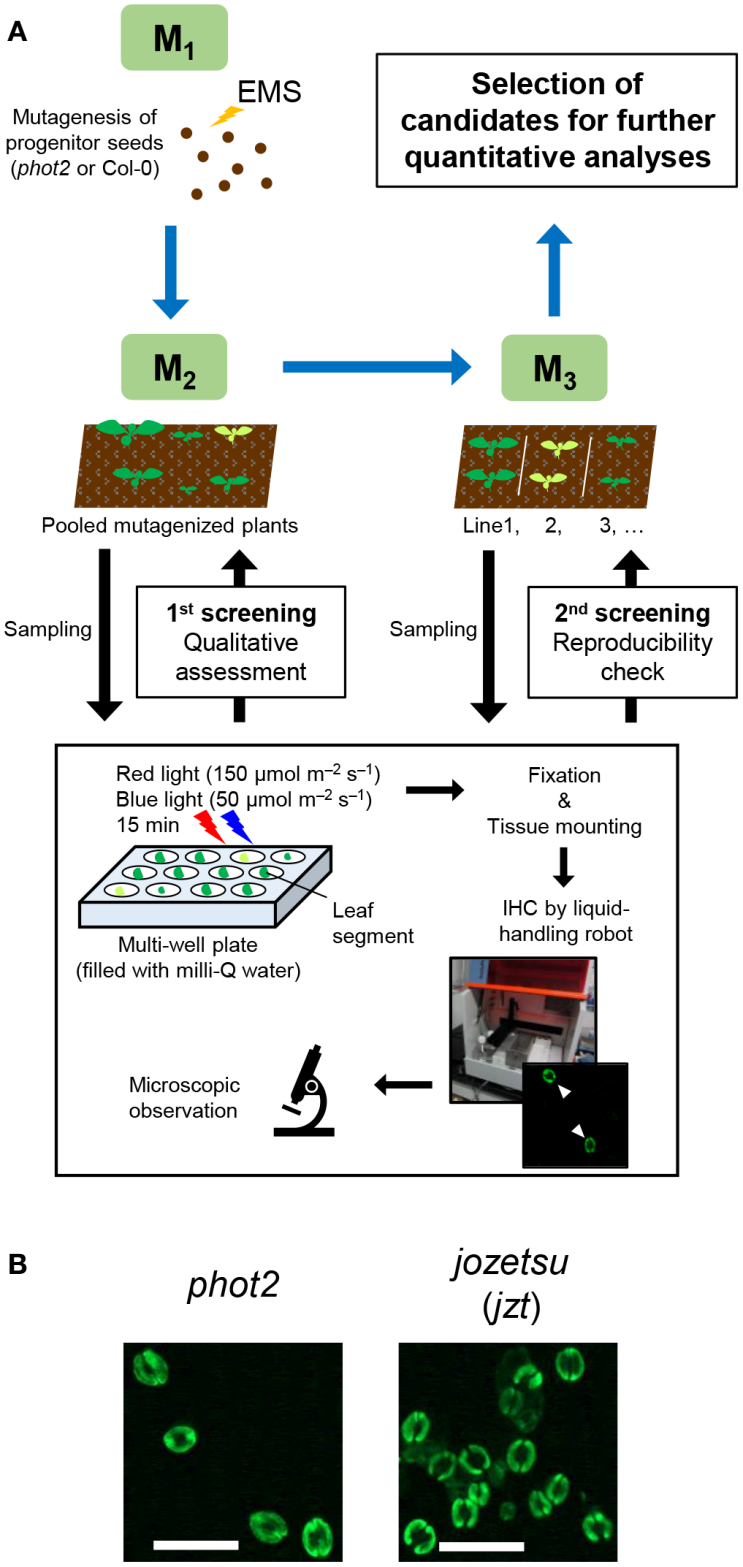
Isolation of *jozetsu* (*jzt*) by immunohistochemical (IHC) screening. Light-induced phosphorylation of the penultimate residue of plasma membrane H^+^-ATPase, threonine, was visualized in guard cells using whole leaves. **(A)** Work-flow of the experiment. **(B)** Typical fluorescence image. Leaves were illuminated with red and blue light (150 and 50 µmol m^–2^ s^–1^, respectively) for 15 min. Scale bar represents 50 µm.

Previously, we developed a novel immunohistochemical technique for visualizing PM H^+^-ATPase and the phosphorylation of its penultimate residue, Thr, in guard cells using whole leaves (Ando and Kinoshita, 2018). Conventional techniques, such as the isolation of guard cell protoplasts (Ueno et al., 2005), require isolation of a sufficient amount of epidermis before the experiments, limiting their application based on plant amount, size, or both. To assess the versatility of the new immunohistochemical technique, we applied it to genetic screening, where plants must be analyzed individually, and thus only single, and sometimes small, leaves are available for the experiment. Utilizing a liquid-handling robot, we successfully semi-automated the process of immunohistochemical visualization of guard-cell PM H^+^-ATPase to prepare many specimens simultaneously. We screened the M_2_ population of EMS-mutagenized *phot2-1* (*phot2*-EMS; about 8,700 plants) and Col-0 (Col-EMS; about 3,200 plants) based on the phosphorylation status of guard-cell PM H^+^-ATPase in leaves illuminated with red (150 µmol m^–2^ s^–1^) and blue light (50 µmol m^–2^ s^–1^) for 15 min (**Figure 1A**). During the experiment, we identified a plant that exhibited increased stomatal density, which was named *jozetsu* (*jzt*) after a Japanese word meaning ‘talkative’ (**Figure 1B**). The successful isolation of plants with stomatal defects demonstrates that our immunohistochemical technique using a leaf works even when the plant material is limited.

### Characterization of *jzt* plant

**Figure 2:**
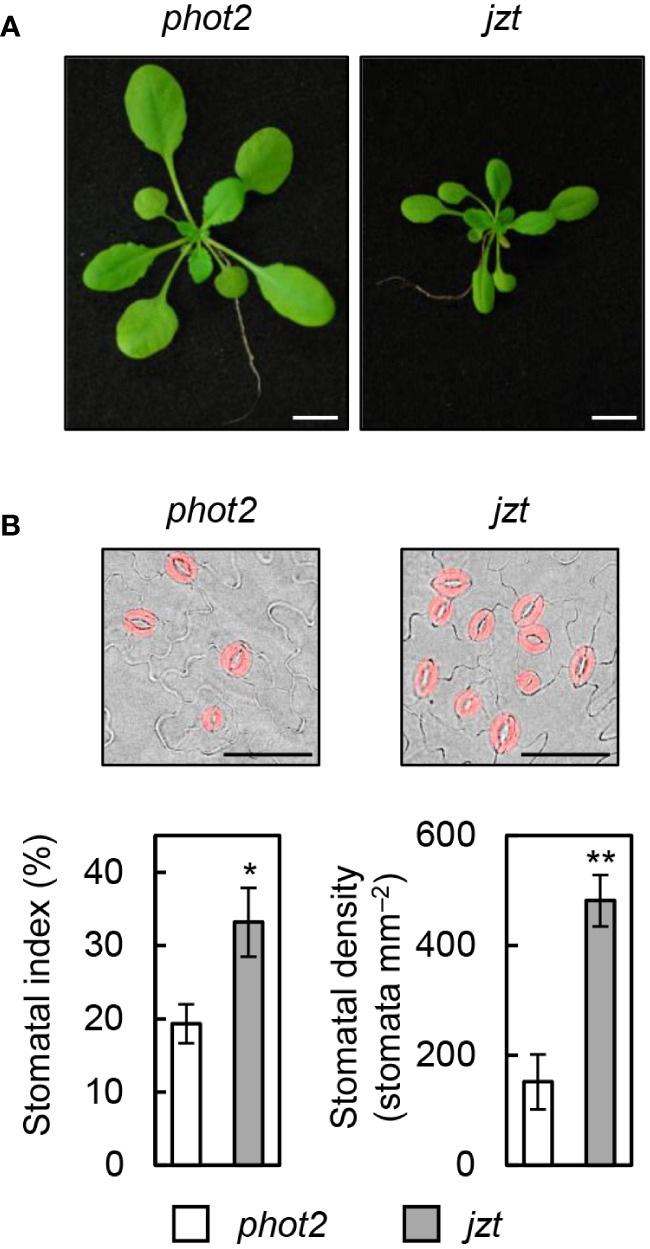
Morphological phenotypes in *jzt*. **(A)** Seedlings of *phot2* and *jzt* at twenty-eight days after sowing (DAS). **(B)** Quantification of the stomatal index and density in *phot2* and *jzt*. Guard cells are indicated by pseudo color (red). Scale bar represents 50 µm. Data represent means of three independent measurements with SDs. Asterisks indicate that the mean of *jzt* is significantly higher than that of *phot2* (one-tailed Student’s t test; ^*^ *P* < 0.01, ^**^ *P* < 0.001).

To identify the responsible mutation in *jzt* plant, we conducted further investigations on *jzt* and characterized this mutant. Initially, we examined whether *jzt* exhibits morphological defects other than the stomatal density. As shown in **Figure 2A**, we observed that *jzt* plants displayed a dwarf phenotype compared to its progenitor *phot2*, suggesting that the putative responsible mutation is involved not only in the stomatal development but also in general plant development. Next, we analyzed the stomatal index and density to quantitatively assess the *jzt* phenotype. In *phot2* leaves, the stomatal index was approximately 20% on average, whereas *jzt* leaves exhibited a 1.5-fold higher stomatal index (**Figure 2B**). Stomatal density in *phot2* and *jzt* was around 150 and 480 stomata mm^–2^, respectively. These results indicate that *jzt* may have a defect in guard-cell differentiation during the stomatal development.

### Identification of the mutation responsible for *jzt* phenotypes

**Figure 3:**
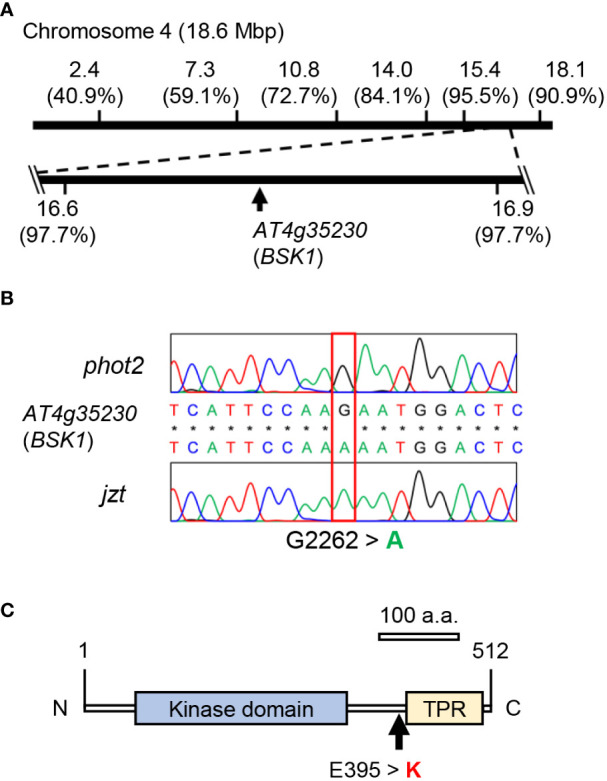
Identification of the mutation in *jzt*. **(A)** Map-based cloning and next-gen sequencing analyses. The mutated gene linked to the stomatal morphology in *jzt* was mapped on the region from 16.6 Mbp to 16.9 Mbp on the chromosome 4, in which *At4g35230* (*BSK1*) has a point mutation. Numbers in the parentheses represent the ratio of non-recombinants. **(B)** Re-sequencing validated a substitution of G2262 to A in *BSK1* gene in *jzt*. **(C)** The mutation in B resulted in the amino acid substitution Glu395 to Lysin (E395 > K) in BSK1. TPR, tetratricopeptide repeat.

We crossed *jzt* with L*er* or *phot2* and conducted map-based cloning or Next-generation sequencing, respectively, to identify the mutation responsible for the *jzt* phenotypes. Map-based cloning revealed that the responsible mutation is located between 16.6 and 16.9 Mb of the chromosome 4 (**Figure 3A**). Through this, we identified single nucleotide substitutions that cause missense mutations in two genes: At4g35150 and At4g35230 encoding *O*-methyltransferase family protein and BSK1, respectively (**Figures 3B, C**; **Supplementary Figures S1A, B**). Since BSK1 is a signaling complex for BR and BR has been shown to be a negative regulator of stomatal development in leaves (Tang et al., 2008; Kim et al., 2012). Public microarray data indicated that *BSK1* transcripts are detectable throughout the entire plant, including leaves, whereas those of *At4g35150* were indicated to express only in the developing embryo (**Supplementary Figure S1C**). These results strongly suggested that the responsible mutation is the substitution (G2262 to A) in BSK1, causing an amino acid change (Glu395 to Lys) at the linker region between the kinase domain and the tetratricopeptide repeat (TPR; **Figure 3C**).

**Figure 4:**
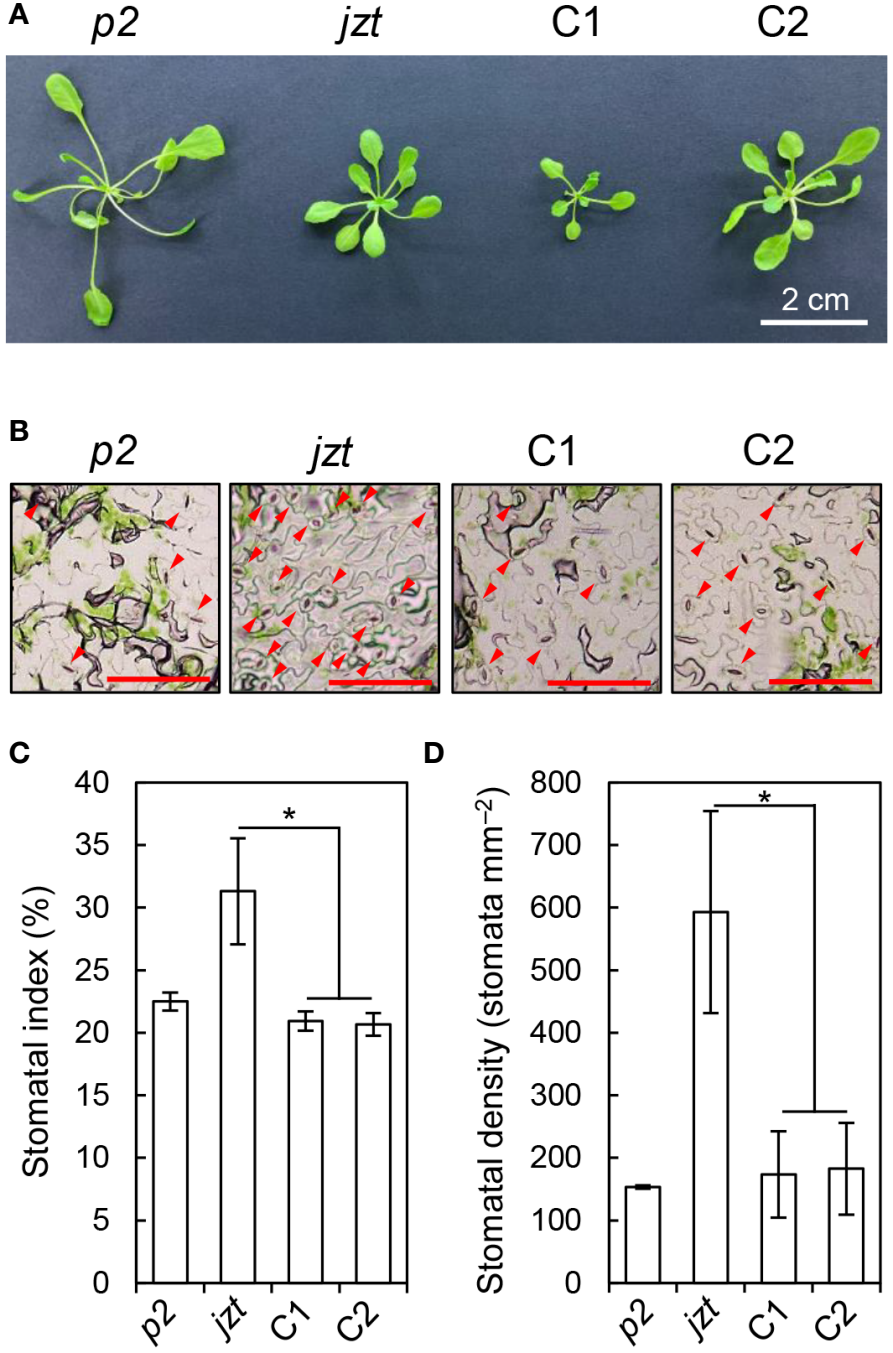
The mutation in BSK1 was responsible for the stomatal morphology in *jzt*. **(A)** Plant phenotype of *phot2* (*p2*), *jzt*, and the complemented lines (C1 and C2). **(B)** Stomatal phenotype of *p2*, *jzt*, C1, and C2. Abaxial epidermis is shown. Arrowheads indicate stomata. Scale bars: 100 µm. **(C, D)** Quantification of the stomatal index **(C)** and density **(D)** in *p2*, *jzt*, C1, and C2. Asterisks indicate that the means of C1 and C2 are significantly lower than that of *jzt* (one-tailed Dunnett’s test; ^*^ *P* < 0.005).

To validate the above results, we transformed *jzt* with a wild-type genomic *BSK1* fragment, including its putative promoter region, and investigated whether the transgene complements the phenotypes in *jzt*. Although the dwarf phenotype was not fully restored in the transgenic lines (**Figure 4A**), they exhibited reduced stomatal index and density compared to *jzt* (**Figures 4B–D**). Therefore, the defect in stomatal development in *jzt* is most likely to be caused by the novel mutation in BSK1.

### The novel mutation in BSK1 exhibits semi-dominant features

**Figure 5:**
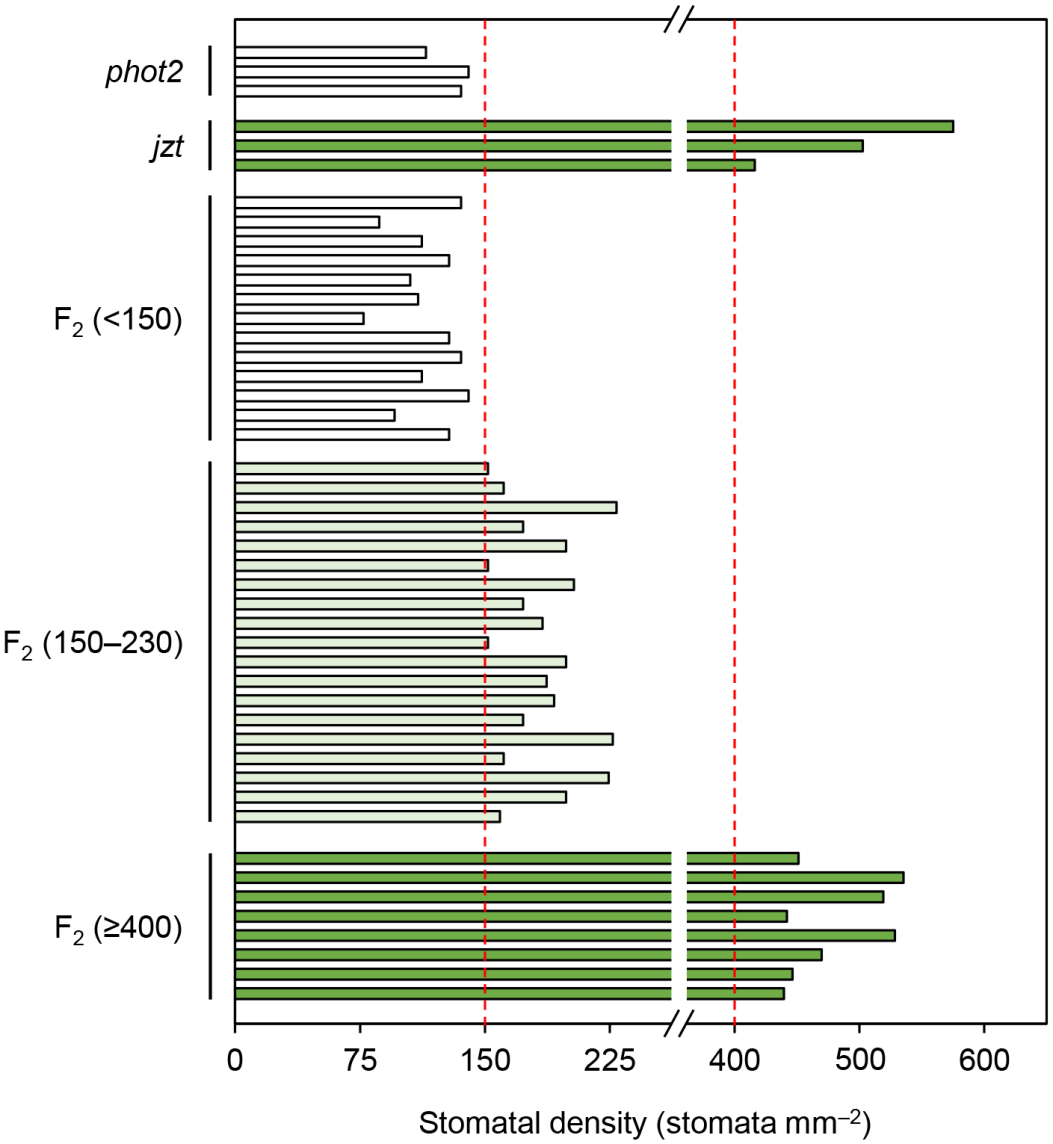
The substitution in *BSK1* in *jzt* was semi-dominant. Stomatal density of the F_2_ population (*n* = 40 plants) obtained from the crossing of *phot2* and *jzt* were analyzed. Three phenotypes were observed: *phot2*-like (stomatal density < 150 stomata mm^–2^), *jzt*-like (>= 400 stomata mm^–2^), and intermediate (150 – 230 stomata mm^–2^). The segregation ratio was 13: 19: 8, which was fitted to the 1: 2: 1 segregation (chi-squared test, χ^2^ = 1.35, *P* = 0.51).

BSKs exhibit functional redundancy, and only the *bsk3-1* mutant shows insensitivity to exogeneous BR treatment (Tang et al., 2008; Sreeramulu et al., 2013). A recent study indicated that a double knock-out of BSK1 and its homolog BSK2 is required to cause the defects in stomatal development similar to those observed in *jtz* (Neu et al., 2019). Consistent with these studies, we could not observe the high stomatal density phenotype in the T-DNA insertion *bsk1* mutant (**Supplementary Figure S2**). These results suggest that the novel mutation in BSK1 identified in this study does not simply result in the functional loss of BSK1 protein. Then, we hypothesized that the mutation is a dominant allele rather than a recessive loss-of-function allele. To test this, we reanalyzed the F_2_ population obtained by crossing *jzt* and *phot2* to quantify the stomatal phenotype in each plant in the population. We confirmed that a quarter of the F_2_ plants exhibited an extremely high stomatal density like *jzt* (> 400 stomata mm^–2^). Another quarter of the population showed *phot2*-like phenotype, where the stomatal density was below 150 stomata mm^–2^. Interestingly, we found that about half of the F_2_ plants showed partially increased stomatal density (150–230 stomata mm^–2^). The segregation ratio of “*phot2*-like”: “intermediate”: “*jzt*-like” was fitted to the well-known 1: 2: 1 autosomal semi-dominant mode of inheritance (**Figure 5**). These results indicate that the novel mutation observed in BSK1 (hereafter referred to *bsk1-4D*) is a semi-dominant allele and thus capable of causing morphological defects by itself.

## Discussion

### Application of the immunohistochemical detection of PM H^+^-ATPase in leaves

The genetic screening conducted in this study is characterized by the direct observation of stomata. Although similar experiments can be found in literature studying guard-cell differentiation (Pillitteri et al., 2007), most previous studies on stomatal physiology have examined phenotypes that indirectly represent plant transpiration through stomata. For example, changes in leaf surface temperature or leaf weight serves as an index for water loss through transpiration (Merlot et al., 2002; Hashimoto et al., 2006; Negi et al., 2008; Tsuzuki et al., 2011; Takemiya et al., 2013a; Tomiyama et al., 2014; Yamauchi et al., 2016). The use of these phenotypes in genetic screening enables the simultaneous handling of multiple samples. On the other hand, immunohistochemical experiment appears to be unsuitable for handling many samples at once, as it involved various treatments with chemical solution as well as washing materials. Recently, however, commercial liquid-handling robots have enabled the automation of immunohistochemistry or *in situ* hybridization experiments (Friml et al., 2003; Matsuzaki et al., 2010). Here, we also demonstrated that the immunohistochemical detection of guard-cell PM H^+^-ATPase can be semi-automated by the robot. Genetic screening, taking advantage of the liquid-handling robot, is ongoing to isolate mutants that exhibit defects in the light-induced phosphorylation of guard-cell PM H^+^-ATPase, which will be reported in a future paper.

Previous techniques for detecting guard-cell PM H^+^-ATPase relied on the isolation of epidermal tissues containing guard cells, and thus, the application is restricted by the availability of epidermis from the plants (Ueno et al., 2005; Hayashi et al., 2011). In this context, the immunohistochemical technique using leaves was expected to enable us to conduct experiments such as genetic screening, where we have to handle individual, and sometimes dwarf, leaves (Ando and Kinoshita, 2018). Successful phenotyping by the immunohistochemistry using a single leaf even in dwarf plants like *jzt*/*bsk1-4D*, demonstrates that our technique has a broad range of application. Thus, it would enable various genetic investigations of guard-cell PM H^+^-ATPase in plants with small and/or few leaves like early-flowering mutants (Kinoshita et al., 2011).

### Novel dominant mutation in BSK1

In this study, we identified a novel semi-dominant mutation in *BSK1* that causes morphological defects, including the overproduction of stomata. Incomplete complementation of the dwarf phenotype in *jzt*/*bsk1-4D* by genomic BSK1 may support that the mutation is a semi-dominant allele (**Figure 4A**). The *BSK* family genes exhibit functional redundancy, and simultaneous knock-out of BSK genes is required to induce the morphological defects (Sreeramulu et al., 2013; Neu et al., 2019). BSK1 is considered one of the substrates of BRI1 in BR signaling (Tang et al., 2008). Crosstalk exists between leucin-rich-repeat receptor-like kinases (LRR RLKs), including BRI1, which regulates the plant growth, development, and innate immunity (Zhu et al., 2013). Previously, the *bsk1-1* mutation was identified as a suppressor mutation for the powdery mildew resistance phenotype in *ENHANCED DISEASE RESISTANCE2*, and BSK1 was shown to associate with FLAGELLIN SENSING2 (FLS2), another LRR RLK that functions in the immune response (Shi et al., 2013). In contrast to the mutation in *jzt*/*bsk1-4D*, *bsk1-1* is a recessive allele that causes a missense mutation in the TPR domain of BSK1 (Shi et al., 2013). TPR was originally identified in yeast and has been considered to be involved in protein–protein interaction (Hirano et al., 1990; Sikorski et al., 1990; Blatch and Lässle, 1999). As *bsk1-1* does not affect the BSK1-FLS2 interaction itself, a functional defect other than the protein interaction may be caused by the *bsk1-1* mutation (Shi et al., 2013). *jzt*/*bsk1-4D* is located in the C-terminal region of the linker between the kinase domain and TPR (**Figure 3C**). Previous reports and the present results imply that the C-terminal region of BSK1, including Glu395 might function in the regulation of or interaction with protein(s) that regulates guard-cell differentiation. The putative target of BSK1 may include other BSK family proteins. Sreeramulu et al. (2013) indicated that loss-of-function of BSK1 restores the insensitivity to 24-epibrassinolide in *bsk3,4,6,7* mutant, suggesting an antagonistic interaction between BSK1 and other BSK family proteins. It would be interesting to investigate the stomatal phenotype in higher-order *bsk* mutants, including *jzt*/*bsk1-4D* mutation. The *jzt*/*bsk1-4D* mutation identified in this study would be a beneficial tool to uncover the protein function of BSK1 in the plant development.

### Perspective

As the phosphorylation of guard-cell PM H^+^-ATPase was detected in *jzt*/*bsk1-4D* (**Figure 1B**), BSK1 may not be necessarily required for light-induced phosphorylation of PM H^+^-ATPase in guard cells. Given that BR signaling has pleiotropic functions (Zhu et al., 2013); however, BSK1 might function in other stomatal response, i.e. immunity (Zeng et al., 2010). Stomatal closure is a part of the immune response to restrict bacterial invasion, whereas the pathogenic bacteria have a mechanism for stomatal reopening to achieve their infection (Melotto et al., 2006). Bacterial invasion also induces a systemic reduction of stomatal density in new leaves emerged after the inoculation (Dutton et al., 2019). Previous studies have revealed a significant role of PM H^+^-ATPase in the plant-pathogen interaction including stomatal movement, in which RPM1-interacting protein 4 (RIN4) function as PM H^+^-ATPase activator for stomatal reopening (Liu et al., 2009; Elmore and Coaker, 2011). RIN4 is a putative phosphorylation target of FLS2, which is likely to be regulated by BSK1 as described above (Shi et al., 2013 **;** Ray et al., 2019). It is noteworthy that BR induces the phosphorylation of PM H^+^-ATPase in the hypocotyl of etiolated seedlings (Minami et al., 2019). These results imply a functional connection between BSK1 and the stomatal immune response. The *jzt*/*bsk1-4D* mutant would also be a useful genetic resource to investigate this hypothesis.

## Data availability statement

The original contributions presented in the study are included in the article/**Supplementary Material**. Further inquiries can be directed to the corresponding authors.

## Author contributions

EA: Conceptualization, Data curation, Formal analysis, Funding acquisition, Investigation, Methodology, Project administration, Supervision, Validation, Visualization, Writing – original draft, Writing – review & editing. KT: Investigation, Writing – original draft. TS: Investigation, Writing – original draft, Methodology, Software. TK: Writing – original draft, Investigation, Methodology, Software, Conceptualization, Data curation, Formal analysis, Funding acquisition, Project administration, Resources, Supervision, Validation, Visualization, Writing – review & editing.

## References

[B1] Akiyama M. Sugimoto H. Inoue S. Takahashi Y. Hayashi M. Hayashi Y.. (2022). Type 2C protein phosphatase clade D family members dephosphorylate guard cell plasma membrane H^+^-ATPase. Plant Physiol. 188, 2228–2240. doi: 10.1093/plphys/kiab571 34894269 PMC8968332

[B2] Ando E. Kinoshita T. (2018). Red light-induced phosphorylation of plasma membrane H+-ATPase in stomatal guard cells. Plant Physiol. 178, 838–849. doi: 10.1104/pp.18.00544 30104254 PMC6181031

[B3] Ando E. Kinoshita T. (2019). Fluence rate dependence of red light-induced phosphorylation of plasma membrane H^+^-ATPase in stomatal guard cell. Plant Signal. Behav. 14, e1561107. doi: 10.1080/15592324.2018.1561107 PMC6351090 30601076

[B4] Ando E. Kollist H. Fukatsu K. Kinoshita T. Terashima I. (2022). Elevated CO_2_ induces rapid dephosphorylation of plasma membrane H^+^-ATPase in guard cells. New Phytol. 236, 2061–2074. doi: 10.1111/nph.18472 36089821 PMC9828774

[B5] Baena G. Xia L. Waghmare S. Yu Z. Guo Y. Blatt M. R.. (2024). Arabidopsis SNARE SYP132 impacts on PIP2;1 trafficking and function in salinity stress. Plant J. doi: 10.1111/tpj.16649 38289468

[B6] Bergmann D. C. Lukowitz W. Somerville C. R. (2004). Stomatal development and pattern controlled by a MAPKK kinase. Science 304, 1494–1497. doi: 10.1126/science.1096014 15178800

[B7] Blatch G. L. Lässle M. (1999). The tetratricopeptide repeat: a structural motif mediating protein-protein interactions. Bioessays 21, 932–939. doi: 10.1002/(SICI)1521-1878(199911)21:11<932::AID-BIES5>3.0.CO;2-N 10517866

[B8] Dutton C. Hõrak H. Hepworth C. Mitchell A. Ton J. Hunt L.. (2019). Bacterial infection systemically suppresses stomatal density. Plant Cell Environ. 42, 2411–2421. doi: 10.1111/pce.13570 31042812 PMC6771828

[B9] Elmore J. M. Coaker G. (2011). The role of the plasma membrane H^+^-ATPase in plant-microbe interaction. Mol. Plant 4, 416–427. doi: 10.1093/mp/ssq083 21300757 PMC3107590

[B10] Friml J. Benková E. Mayer U. Palme K. Muster G. (2003). Automated whole mount localisation techniques for plant seedlings. Plant J. 34, 115–124. doi: 10.1046/j.1365-313x.2003.01705.x 12662314

[B11] Fuglsang A. T. Palmgren M. (2021). Proton and calcium pumping P-type ATPases and their regulation of plant responses to the environment. Plant Physiol. 187, 1856–1875. doi: 10.1093/plphys/kiab330 35235671 PMC8644242

[B12] Han S. K. Kwak J. M. Qi X. (2021). Stomatal lineage control by developmental program and environmental cues. Front. Plant Sci. 12. doi: 10.3389/fpls.2021.751852 PMC8542704 34707632

[B16] Hashimoto M. Negi J. Young J. Israelsson M. Shroeder J. I. Iba K. (2006). Arabidopsis HT1 kinase controls stomatal movements in response to CO_2_ . Nat. Cell Biol. 8, 391–397. doi: 10.1038/ncb1387 16518390

[B15] Hayash Y. Nakamura S. Takemiya A. Takahashi Y. Shimazaki K. Kinoshita T. (2010). Biochemical characterization of *in vitro* phosphorylation and dephosphorylation of the plasma membrane H^+^-ATPase. Plant Cell Physiol. 51, 1186–1196. doi: 10.1093/pcp/pcq078 20516032

[B14] Hayashi M. Inoue S. Takahashi K. Kinoshita T. (2011). Immunohistochemical detection of blue light-induced phosphorylation of the plasma membrane H^+^-ATPase in stomatal guard cells. Plant Cell Physiol. 52, 1238–1248. doi: 10.1093/pcp/pcr072 21666226

[B13] Hayashi M. Inoue S. Ueno Y. Kinoshita T. (2017). A Raf-like protein kinase BHP mediates blue light-dependent stomatal opening. Sci. Rep. 7, 45586. doi: 10.1038/srep45586 28358053 PMC5372365

[B17] He J. X. Gendron J. M. Yang Y. Li J. Wang Z. Y. (2002). The GSK3-like kinase BIN2 phosphorylates and destabilizes BZR1, a positive regulator of the brassinosteroid signaling pathway in Arabidopsis. Proc. Natl. Acad. Sci. U.S.A. 99, 10185–10190. doi: 10.1073/pnas.152342599 12114546 PMC126645

[B18] Hirano T. Kinoshita N. Morikawa K. Yanagida M. (1990). Snap helix with knob and hole: essential repeats in S. pombe nuclear protein nuc2^+^ . Cell 60, 319–328. doi: 10.1016/0092-8674(90)90746-2 2297790

[B1000] Hothorn T. Bretz F. Westfall P. (2008). Simultaneous inference in general parametric models. Biom. J. 50, 346–363. doi: 10.1002/bimj.200810425 18481363

[B19] Inoue S. Kinoshita T. (2017). Blue light regulation of stomatal opening and the plasma membrane H^+^-ATPase. Plant Physiol. 174, 531–538. doi: 10.1104/pp.17.00166 28465463 PMC5462062

[B20] Jezek M. Blatt M. R. (2017). The membrane transport system of the guard cell and its integration for stomatal dynamic. Plant Physiol. 174, 487–519. doi: 10.1104/pp.16.01949 28408539 PMC5462021

[B21] Kanaoka M. M. Pillitteri L. J. Fujii H. Yoshida Y. Bogenschutz N. L. Takabayashi. (2008). SCREAM/ICE1 and SCREAM2 specify three cell-state transitional steps leading to Arabidopsis stomatal differentiation. Plant Cell 20, 1775–1785. doi: 10.1105/tpc.108.060848 18641265 PMC2518248

[B22] Kang C. Y. Lian H. L. Wang F. F. Huang J. R. Yang H. Q. (2009). Cryptochromes, phytochromes, and COP1 regulate light-controlled stomatal development in Arabidopsis. Plant Cell 21, 2624–2641. doi: 10.1105/tpc.109.069765 19794114 PMC2768914

[B23] Kim T. W. Guan S. Burlingame A. L. Wang Z. Y. (2011). The CDG1 kinase mediates brassinosteroid signal transduction from BRI1 receptor kinase to BSU1 phosphatase and GSK3-like kinase BIN2. Mol. Cell 43, 561–571. doi: 10.1016/j.molcel.2011.05.037 21855796 PMC3206214

[B24] Kim T. W. Michniewicz M. Bergmann D. C. Wang Z. Y. (2012). Brassinosteroid regulates stomatal development by GSK3-mediated inhibition of a MAPK pathway. Nature 482, 419–422. doi: 10.1038/nature10794 22307275 PMC3292258

[B25] Kinoshita T. Caño-Delgado A. Seto H. Hiranuma S. Fujioka S. Yoshida S.. (2005). Binding of brassinosteroids to the extracellular domain of plant receptor kinase BRI1. Nature 433, 167–171. doi: 10.1038/nature03227 15650741

[B26] Kinoshita T. Ono N. Hayashi Y. Morimoto S. Nakamura S. Soda M.. (2011). *FLOWERING LOCUS T* regulates stomatal opening. Curr. Biol. 21, 1232–1238. doi: 10.1016/j.cub.2011.06.025 21737277

[B27] Kinoshita T. Shimazaki K. (1999). Blue light activates the plasma membrane H^+^-ATPase by phosphorylation of the C-terminus in stomatal guard cells. EMBO J. 18, 5548–5558. doi: 10.1093/emboj/18.20.5548 10523299 PMC1171623

[B28] Kinoshita T. Shimazaki K. (2002). Biochemical evidence for the requirement of 14-3-3 protein binding in activation of the guard-cell plasma membrane H^+^-ATPase by blue light. Plant Cell Physiol. 43, 1359–1365. doi: 10.1093/pcp/pcf167 12461136

[B29] Lawson T. Matthews J. (2020). Guard cell metabolism and stomatal function. Annu. Rev. Plant Biol. 71, 273–302. doi: 10.1146/annurev-arplant-050718-100251 32155341

[B30] Li J. Chory J. (1997). A putative leucin-rich repeat receptor kinase involved in brassinosteroid signal transduction. Cell 90, 929–938. doi: 10.1016/S0092-8674(00)80357-8 9298904

[B31] Li J. Wen J. Lease K. A. Doke J. T. Tax F. ,. E. Walker J. C. (2002). BAK1, an *Arabidopsis* LRR receptor-like protein kinase, interacts with BRI1 and modulates brassionosteroid signaling. Cell 110, 213–222. doi: 10.1016/S0092-8674(02)00812-7 12150929

[B32] Lightner J. Caspar T. (1998). Seed mutagenesis of arabidopsis. Methods Mol. Biol. 82, 91–103. 9664417

[B33] Liu J. Elmore J. M. Fuglsang A. T. Palmgren M. G. Staskawicz B. J. Coaker G. (2009). RIN4 functions with plasma membrane H^+^-ATPases to regulate stomatal apertures during pathogen attack. PloS Biol. 7, e1000139. doi: 10.1371/journal.pbio.1000139 19564897 PMC2694982

[B34] MacAlister C. A. Ohashi-Ito K. Bergmann D. C. (2007). Transcription factor control of asymmetric cell divisions that establish the stomatal lineage. Nature 445, 537–540. doi: 10.1038/nature05491 17183265

[B35] Matsuzaki Y. Ogawa-Ohnishi M. Mori A. Matsubayashi Y. (2010). Secreted peptide signals required for maintenance of root stem cell niche in Arabidopsis. Science 329, 1065–1067. doi: 10.1126/science.1191132 20798316

[B36] Melotto M. Underwood W. Koczan J. Nomura K. He S. Y. (2006). Plant stomata function in innate immunity against bacterial invasion. Cell 126, 969–980. doi: 10.1016/j.cell.2006.06.054 16959575

[B37] Merlot S. Mustilli A. C. Genty B. North H. Lefebvre V. Sotta B.. (2002). Use of infrared thermal imaging to isolate Arabidopsis mutants defective in stomatal regulation. Plant J. 30, 601–609. doi: 10.1046/j.1365-313x.2002.01322.x 12047634

[B38] Minami A. Takahashi K. Inoue S. Tada Y. Kinoshita T. (2019). Brassinosteroid induces phosphorylation of the plasma membrane H^+^-ATPase during hypocotyl elongation in *Arabidopsis thaliana* . Plant Cell Physiol. 60, 935–944. doi: 10.1093/pcp/pcz005 30649552

[B39] Nam K. H. Li J. (2002). BRI1/BAK1 a receptor kinase pair mediating brassinosteroid signaling. Cell 110, 203–212. doi: 10.1016/S0092-8674(02)00814-0 12150928

[B40] Negi J. Matsuda O. Nagasawa T. Oba Y. Takahashi H. Kawai-Yamada M.. (2008). CO_2_ regulator SLAC1 and its homologues are essential for anion homeostasis in plant cells. Nature 452, 483–486. doi: 10.1038/nature06720 18305482

[B41] Neu A. Eilbert E. Asseck L. Y. Slane D. Henschen A. Wang K.. (2019). Constitutive signaling activity of a receptor-associated protein links fertilization with embryonic patterning in *Arabidopsis thaliana.* Proc. Natl. Acad. Sci. U.S.A. 116, 5795–5804. doi: 10.1073/pnas.1815866116 PMC6431185 30833400

[B42] Ohashi-Ito K. Bergmann D. C. (2006). Arabidopsis FAMA controls the final proliferation/differentiation switch during stomatal development. Plant Cell 18, 2493–2505. doi: 10.1105/tpc.106.046136 17088607 PMC1626605

[B43] Palmgren M. (2023). P-type ATPases: Many more enigmas left to solve. J. Biol. Chem. 299, 105352. doi: 10.1016/j.jbc.2023.105352 37838176 PMC10654040

[B44] Pillitteri L. J. Sloan D. Bogneschutz N. L. Torii K. U. (2007). Termination of asymmetric cell division and differentiation of stomata. Nature 445, 501–505. doi: 10.1038/nature05467 17183267

[B45] Qi X. Torii K. U. (2018). Hormonal and environmental signals guiding stomatal development. BMC Biol. 16, 21. doi: 10.1186/s12915-018-0488-5 29463247 PMC5819259

[B47] Ray S. K. Macoy D. M. Kim W. Y. Lee S. Y. (2019). Role of RIN4 in regulating PAMP-triggered immunity and effector-triggered immunity: current status and future perspectives. Mol. Cells 42, 503–511. doi: 10.14348/molcells.2019.2433 31362467 PMC6681865

[B46] R Core Team (2023). R: A language and environment for statistical computing (Vienna: R foundation for statistical computing). Available at: https://www.R-project.org/.

[B48] Roelfsema M. R. G. Hedrich R. Geiger D. (2012). Anion channels: master switches of stress responses. Trends Plant Sci. 17, 221–229. doi: 10.1016/j.tplants.2012.01.009 22381565

[B49] Shi H. Shen Q. Qi Y. Yan H. Nie H. Chen Y.. (2013). BR-SIGNALING KINASE1 physically associates with FLAGELLIN SENSING2 and regulates plant innate immunity in Arabidopsis. Plant Cell 25, 1143–1157. doi: 10.1105/tpc.112.107904 23532072 PMC3634682

[B50] Shimazaki K. Doi M. Assmann S. M. Kinoshita T. (2007). Light regulation of stomatal movement. Annu. Rev. Plant Biol. 58, 219–247. doi: 10.1146/annurev.arplant.57.032905.105434 17209798

[B51] Sikorski R. S. Boguski M. S. Goebl M. Hieter P. (1990). A repeating amino acid motif in CDC23 defines a family of proteins and a new relationship among genes required for mitosis and RNA synthesis. Cell 60, 307–317. doi: 10.1016/0092-8674(90)90745-z 2404612

[B52] Sreeramulu S. Mostizky Y. Sunitha S. Shani E. Nahum H. Salomon D.. (2013). BSKs are partially redundant positive regulators of brassinosteroid signaling in Arabidopsis. Plant J. 74, 905–919. doi: 10.1111/tpj.12175 23496207

[B53] Suzuki T. Kawai T. Takemura S. Nishiwaki M. Suzuki T. Nakamura K.. (2018). Development of the Mitsucal computer system to identify causal mutation with a high-throughput sequencer. Plant Reprod. 31, 117–128. 29497825 10.1007/s00497-018-0331-8

[B54] Takemiya A. Sugiyama N. Fujimoto H. Tsutsumi T. Yamauchi S. Hiyama A.. (2013a). Phosphorylation of BLUS1 kinase by phototropins is a primary step in stomatal opening. Nat. Commun. 4, 2094. doi: 10.1038/ncomms3094 23811955

[B55] Takemiya A. Yamauchi S. Yano T. Ariyoshi C. Shimazaki K. (2013b). Identification of a regulatory subunit of protein phosphatase 1 which mediates blue light signaling for stomatal opening. Plant Cell Physiol. 54, 24–35. doi: 10.1093/pcp/pcs073 22585556

[B56] Tang W. Kim T. W. Oses-Prieto J. A. Sun Y. Deng Z. Zhu S.. (2008). BSKs mediate signal transduction from the receptor kinase BRI1 in *Arabidopsis* . Science 321, 557–560. doi: 10.1126/science.1156973 18653891 PMC2730546

[B57] Tomiyama M. Inoue S. Tsuzuki T. Soda M. Morimoto S. Okigaki Y.. (2014). Mg-chelatase I subunit 1 and Mg-protoporphyrin IX methyltransferase affect the stomatal aperture in *Arabidopsis thaliana* . J. Plant Res. 127, 553–563. doi: 10.1007/s10265-014-0636-0 24840863 PMC4683165

[B58] Tsuzuki T. Takahashi K. Inoue S. Okigaki Y. Tomiyama M. Hossain M. A.. (2011). Mg-chelatase H subunit affects ABA signaling in stomatal guard cells, but is not an ABA receptor in *Arabidopsis thaliana* . J. Plant Res. 124, 527–538. doi: 10.1007/s10265-011-0426-x 21562844 PMC3129500

[B59] Ueno K. Kinoshita T. Inoue S. Emi T. Shimazaki K. (2005). Biochemical characterization of plasma membrane H+-ATPase activation in guard cell protoplasts of Arabidopsis thaliana in response to blue light. Plant Cell Physiol. 46, 955–963. doi: 10.1093/pcp/pci104 15821287

[B60] Wang X. Chory J. (2006). Brassinosteroids regulate dissociation of BKI1, a negative regulator of BRI1 signaling, from the plasma membrane. Science 313, 1118–1122. doi: 10.1126/science.1127593 16857903

[B61] Wang X. Kota U. He K. Blackburn K. Li J. Goshe M. B.. (2008). Sequential transphosphorylation of the BRI1/BAK1 receptor kinase complex impacts early events in brassionsteroid signaling. Dev. Cell 15, 220–235. doi: 10.1016/j.devcel.2008.06.011 18694562

[B62] Wang Z. Y. Nakano T. Gendron J. He J. Chen M. Vafeados D.. (2002). Nuclear-localized BZR1 mediates brassinosteroid-induced growth and feedback suppression of brassinosteroid biosynthesis. Dev. Cell. 2, 505–513. doi: 10.1016/s1534-5807(02)00153-3 11970900

[B63] Wong J. H. Klejchová M. Snipes S. A. Nagpal P. Bak G. Wang B.. (2021). SAUR proteins and PP2C.D phosphatases regulate H^+^-ATPases and K^+^ channels to control stomatal movements. Plant Physiol. 185, 256–273. doi: 10.1093/plphys/kiaa023 33631805 PMC8133658

[B64] Xia L. Marquès-Bueno M. M. Bruce C. G. Karnik R. (2019). Unusual roles of secretory SNARE SYP132 in plasma membrane H^+^-ATPase traffic and vegetative plant growth. Plant Physiol. 180, 837–858. doi: 10.1104/pp.19.00266 30926657 PMC6548232

[B65] Xue Y. Yang Y. Yang Z. Wang X. Guo Y. (2018). VAMP711 is required for abscisic acid-mediated inhibition of plasma membrane H^+^-ATPase activity. Plant Physiol. 178, 1332–1343. doi: 10.1104/pp.18.00499 30217827 PMC6236615

[B66] Yamauchi S. Takemiya A. Sakamoto T. Kurata T. Tsutsumi T. Kinoshita T.. (2016). The plasma membrane H^+^-ATPase AHA1 plays a major role in stomatal opening in response to blue light. Plant Physiol. 171, 2731–2743. doi: 10.1104/pp.16.01581 27261063 PMC4972258

[B67] Yin Y. Wang Z. Y. Mora-Garcia S. Li J. Yoshida S. Asami T.. (2002). BES1 accumulates in the nucleus in response to brassinosteroids to regulate gene expression and promote stem elongation. Cell 109, 181–191. doi: 10.1016/s0092-8674(02)00721-3 12007405

[B68] Zeng W. Melotto M. He S. Y. (2010). Plant stomata: a checkpoint of host immunity and pathogen virulence. Curr. Opin. Biotechnol. 21, 599–603. doi: 10.1016/j.copbio.2010.05.006 20573499 PMC2946497

[B69] Zhu J. Y. Sae-Seaw J. Wang Z. Y. (2013). Brassinosteroid signalling. Development 140, 1615–1620. doi: 10.1242/dev.060590 23533170 PMC3621480

