# Supplementary material for: A novel semi-dominant mutation in brassinosteroid signaling kinase1 increases stomatal density
Source: Front Plant Sci. 2024 Apr 2;15:1377352. doi: 10.3389/fpls.2024.1377352 (PMC11019013; doi:10.3389/fpls.2024.1377352)
Supplement: Supplementary file 1 [file DataSheet_1.pdf]

Supplemental Figure S1

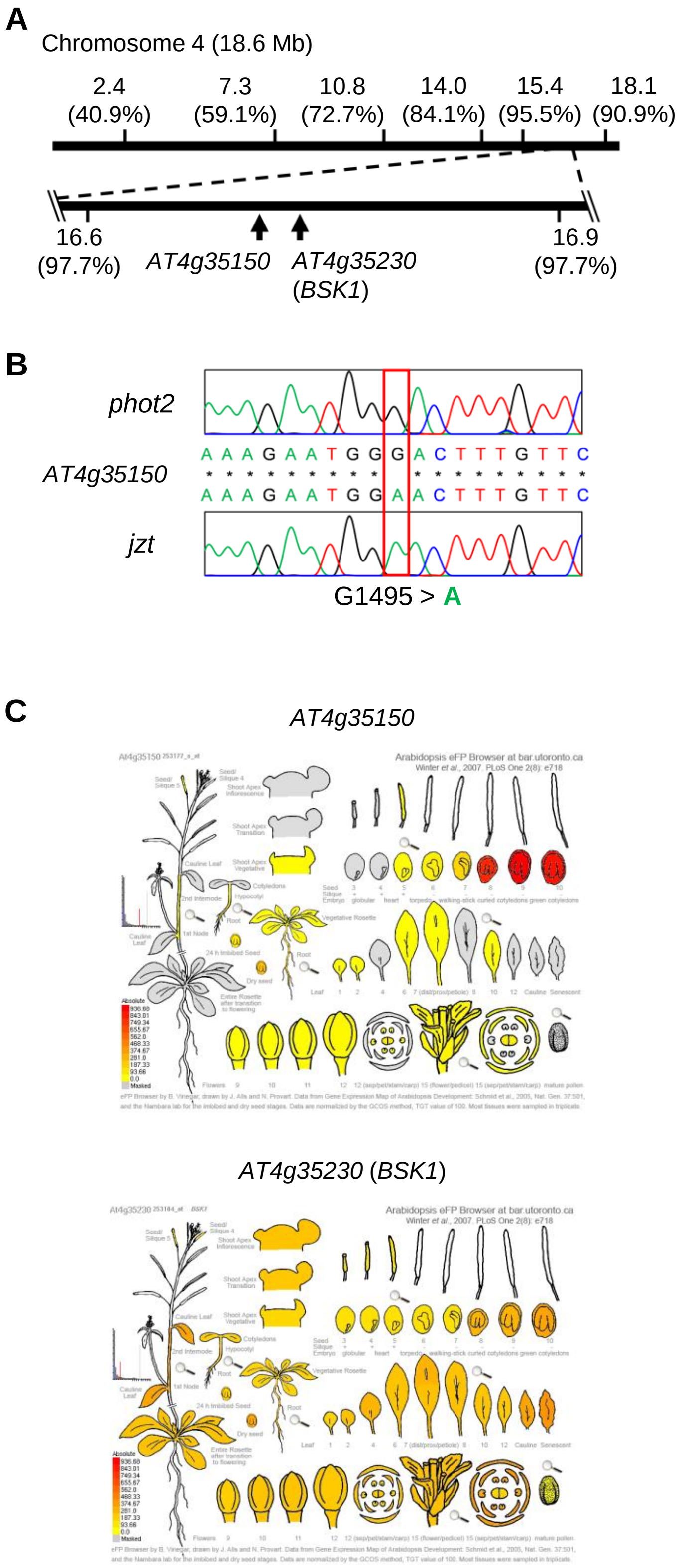

Supplemental Figure S1. Additional mutation in *jzt*. (A and B) At4g35150 also has a substitution (G1495A). (C) Expression profiles of *At4g35150* (upper) and *BSK1* (lower). Data and images were obtained from Arabidopsis eFP browser.

Supplemental Figure S2

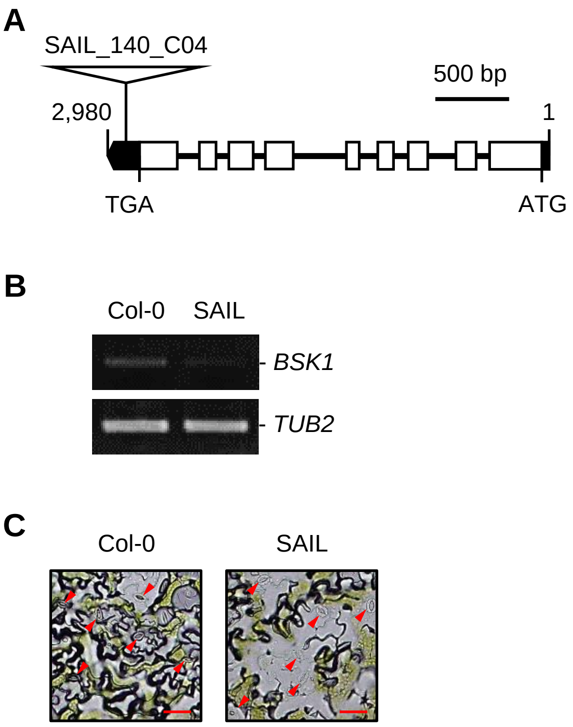

Supplemental Figure S2. Knock-down of *BSK1* did not affect stomatal density. (A) Schematic diagram of T-DNA (SAIL_140_C04) insertion point. (B) RT-PCR experiment for BSK1 expression in Col-0 and the mutant (SAIL). (C) Microscopic images representing the isolated epidermis from Col-0 and SAIL. Arrowheads indicate stomata. Scale bars: 50 µm.

Supplemental Table S1. List of primer sets used in this study.

Primer	Sequence (5' to 3')	Usage
BSK1-Seq-Fwd	ACTCCCAATCTTGACCGAGTTC	Sequencing of G2212 in *BSK1*
BSK1-Seq-Rev	GACTTCGCCGACCAAATACAG	
bsk1-SAIL-LP	GACTGCAACGAGATGAATTCC	T-DNA insertion check
bsk1-SAIL-RP	TCTCCAACTGTATTTGGTCGG	
BSK1-Fwd1	TTCCAATCATTAGCCTGGCCAC	(1st PCR) Vector construction
BSK1-Rev1	AGCAAGCATTATTGTCCTTAAGTCTTG	
BSK1-Fwd2	CCATGATTACGAATTACTTCACTCTTTGCCTTTTCTACAC	(2nd PCR) Vector construction
BSK1-Rev2	TACCGAGCTCGAATTTATGTCTTCAACCGTCTCTAATCTC	
RT-*BSK1*-Fwd	CATTTGTTCCATTGGGAAAATCAGAC	RT-PCR
RT-*BSK1*-Rev	CAACGTCAATGAACTGTGAGTAACAG	
RT-*TUB2*-Fwd	CATTGTTGATCTCTAAGATCCGTG	RT-PCR
RT-*TUB2*-Rev	TACTGCTGAGAACCTCTTGAG	

Underlined sequences represent 15-bp overlaps homologous to each end of EcoRI-digested pCAMBIA1300, which are required for In-Fusion cloning.
